# An adaptive association test for microbiome data

**DOI:** 10.1186/s13073-016-0302-3

**Published:** 2016-05-19

**Authors:** Chong Wu, Jun Chen, Junghi Kim, Wei Pan

**Affiliations:** Division of Biostatistics, University of Minnesota, 420 Delaware St. SE, Minneapolis, 55455 USA; Division of Biomedical Statistics and Informatics, Department of Health Sciences Research, Mayo Clinic, 200 First St. SW, Rochester, 55905 USA

## Abstract

There is increasing interest in investigating how the compositions of microbial communities are associated with human health and disease. Although existing methods have identified many associations, a proper choice of a phylogenetic distance is critical for the power of these methods. To assess an overall association between the composition of a microbial community and an outcome of interest, we present a novel multivariate testing method called aMiSPU, that is joint and highly adaptive over all observed taxa and thus high powered across various scenarios, alleviating the issue with the choice of a phylogenetic distance. Our simulations and real-data analyses demonstrated that the aMiSPU test was often more powerful than several competing methods while correctly controlling type I error rates. The R package MiSPU is available at https://github.com/ChongWu-Biostat/MiSPU and CRAN.

**Electronic supplementary material:**

The online version of this article (doi:10.1186/s13073-016-0302-3) contains supplementary material, which is available to authorized users.

## Background

A variety of microbial communities (i.e., microbiotas) and their genomes (i.e., microbiome) exist throughout the human body [1] and play an important role in one’s overall health, such as food digestion, nutrition, development and regulation of the immune system, and prevention of the invasion and growth of pathogens [2]. On the other hand, disruptions of the human microbial communities are associated with a wide range of human diseases, such as liver cancer [3], obesity [4], colorectal cancer [5], inflammatory bowel disease (IBD) [6], type 2 diabetes [7], and antibiotic-associated diarrhea [8]. Understanding the association between human microbiotas and diseases might help in diagnosing disease and developing personalized medicine [9] that restores a disturbed microbial ecosystem to a healthy state, for instance, using a personalized synthetic community and complementary set of nutrients [2].

Recent advances in sequencing technologies have made it feasible to profile microbiotas in a large number of samples via targeted sequencing of the 16S rRNA gene [10], and extend the study of the human genome to the human microbiome, which consists of the collection of the microbial genomes at various sites of the human body and is seen as an extended human genome [11]. Many human microbiome studies aim to detect a possible association of the human microbiome with a phenotype, such as a disease status, called an outcome (of interest) here, after adjusting for potential confounders. These association studies not only can improve our understanding of the non-genetic components of complex traits and diseases, but also might open up an entirely new way for drug development. Although univariate tests (on a single taxon one by one) are widely used in the analysis of differential abundance, multivariate tests (on multiple taxa jointly and simultaneously) have become increasingly popular due to their higher statistical power in aggregating multiple weak associations and reducing the burden of multiple testing. Furthermore, many univariate tests critically depend on some strong parametric assumptions on the distributions or mean-variance functional forms for microbiome data, leading to inflated type I errors when the assumptions are violated [12]. In contrast, no such assumption is imposed in our proposed multivariate test, which, coupled with a proposed permutation procedure for *p* value calculation, is essentially semi-parametric and applicable to even small sample size problems. In this paper, we mainly focus on multivariate tests.

One popular method for testing the association between an overall microbiome composition and an outcome of interest is to use a distance- or dissimilarity-based test, such as PERMANOVA [13]. Via the standard pipelines such as QIIME and mothur [14, 15], the 16S sequence tags are usually clustered into operational taxonomic units (OTUs), which can be considered surrogates for biological taxa within a specified amount of sequence divergence allowed for each OTU. At 97 % similarity, these OTUs represent common species. A specific distance measure is chosen to measure the dissimilarity between each pair of samples, taking into account the phylogeny among taxa. Then the pairwise distance is compared to the distribution of the outcome of interest for evaluating the association between the overall microbiome composition and the outcome. Recently, a new method called the microbiome regression-based kernel association test (MiRKAT) was proposed [16]. Incorporating phylogenetic relationships among taxa, MiRKAT transforms a phylogenetic distance metric into a kernel to measure similarities among samples. Then a semi-parametric kernel machine regression framework is applied to evaluate the association. MiRKAT allows for an easy covariate adjustment and extensions to other types of outcome. By the correspondence between the distance-based association testing and kernel machine regression [16, 17], MiRKAT is closely related to distance-based methods, such as PERMANOVA. In addition, MiRKAT provides an omnibus test that combines several relevant kernels making it more robust across different scenarios. However, the choice of kernels has to be decided by the end user, and more importantly, no automatic taxon selection or weighting is implemented in the framework.

Up till now, numerous distance measures have been developed to depict community differences between two samples. Among many possible distance metrics, the UniFrac-type distance metrics are most popular. They account for phylogenetic relationships among microbial taxa [18–20]. There are several different versions of UniFrac. The unweighted UniFrac distance [18], which is defined as the fraction of the branch length of the tree that leads to descendants from either sample, but not both, is a qualitative diversity measure and is very efficient in detecting abundance changes in rare taxa given that more prevalent species are likely to be present in all individuals. In contrast, the weighted UniFrac distance [19], which weights the branches of a phylogenetic tree based on the abundance differences, is more sensitive to changes in abundant taxa. The generalized UniFrac distance [20] was introduced to unify the weighted and unweighted versions by striking a balance in weighting between relative differences and absolute differences. Many other distances ignoring phylogenetic information are also available. The Bray–Curtis distance [21], for example, quantifies the taxonomic dissimilarity between two samples on the basis of the OTU counts only.

Noise accumulation is a vital problem for high-dimensional data. For example, due to noise accumulation in estimating the population centroids in a high-dimensional feature space, classification using all features can be as bad as a random guess [22]. A severe limitation of kernel- or distance-based methods is that they do not conduct variable selection or variable weighting, which can alleviate noise accumulation and is crucial for high-dimensional microbiome data. In particular, with the dimension much larger than the sample size, some and even most microbial taxa may not be associated with the outcome. Without variable selection or weighting, using all the taxa for distance or kernel calculations simply contributes noise, leading to power loss as to be shown. Therefore, differential weighting of the microbial taxa according to their importance can potentially improve the power of a microbiome association test. We, thus, propose a data-driven approach to achieve adaptive weighting of the taxa based on the data. The proposed method is based on a generalized taxon proportion combining microbial abundance information with phylogenetic tree information and an adaptive test called the aSPU test, which is based on a family of a sum of powered score (SPU) tests [23], incorporating variable weighting. Each SPU test is indexed as SPU(*γ*) by an integer *γ*>0 that controls the extent of weighting on the variables. We call the corresponding tests MiSPU(*γ*) (microbiome-based sum of powered score) and aMiSPU (adaptive MiSPU) when applied to microbiome data. We will demonstrate through numerical simulations and analysis of real data that aMiSPU can be easily applied and is much more powerful than existing tests in most scenarios with well-controlled type I error rates. Although aMiSPU was inspired by the aSPU test, the two differ in whether and how to accommodate unique features of microbial data. In particular, we propose the generalized taxon proportion to combine microbial abundance information and phylogenetic tree information simultaneously. As shown in numerical simulations, directly applying the aSPU test with OTU abundances generally failed to achieve high power. Finally, an R package MiSPU that implements MiSPU with a C++ version of UniFrac distance calculation has been developed.

## Methods

### Data and notation

Suppose *n* samples have been collected, each with a microbial community profile. For sample *i*, let *Y*_*i*_ denote an outcome of interest, which can be binary (e.g., disease status) or continuous. Let *X*_*i*_=(*X*_*i*1_,…,*X*_*ip*_) be the *p* covariates, such as age, gender, and other clinical and environmental variables that we want to adjust for. Let *Z*_*i*_=(*Z*_*i*1_,…,*Z*_*im*_) be the abundances of *m* taxa derived from the observed *q* OTUs for the *i*th sample. Note that an OTU represents a common species while a taxon is a group of one or more species. Here, we assume that each of *Z*_*i*1_,…,*Z*_*iq*_ is the count of the OTU in sample *i* and *Z*_*ik*_, *q*+1≤*k*≤*m*, is the sum of the counts of the OTUs belonging to taxon *k* in sample *i*. The evolutionary relationships among these OTUs and taxa are given by a rooted phylogenetic tree, which contains all *q* OTUs (as leaf nodes) and *m*−*q* taxa (as internal nodes). Suppose *b*_*k*_ is the distance from the root of the phylogenetic tree to taxon *k*, and $p_{ik}=Z_{ik} / \sum _{j=1}^{q}Z_{ij}$ is the proportion of taxon *k* in sample *i*. The goal is to test for a possible association between the overall microbial community composition and the outcome of interest after adjusting for the covariates.

### A new class of tests: MiSPU

**Fig. 1:**
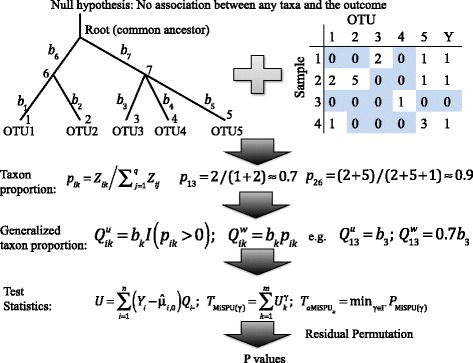
Schematic description of the use and steps in aMiSPU. Input data consist of a rooted phylogenetic tree, a sample of OTU counts, an outcome of interest, and possibly some covariates. *OTU* operational taxonomic unit

The MiSPU and aMiSPU tests are introduced in this section. Figure 1 illustrates the overall structure of the tests, detailing the input (a rooted phylogenetic tree, a sample of OTU counts, an outcome of interest, and possibly some covariates) and the three key steps: calculating a generalized taxon proportion for each taxon, calculating the test statistics, and applying a residual permutation scheme to obtain the *p* values.

One major characteristic of microbial composition data is that taxa are related as described by a phylogenetic tree. Phylogenetic distance measures that account for phylogenetic relationships among taxa can be much more powerful than those ignoring evolutionary information [20]. Among these, UniFrac distances are most popular. Consider two samples *i* and *j*. The unweighted UniFrac distance, which considers only species presence or absence, is a qualitative measure and is defined as [18]:

$$\begin{array}{*{20}l} d_{ij}^{U} =\frac{\sum_{k = 1}^{m}\{b_{k} |I(p_{ik}>0)-I(p_{jk}>0)|\}}{\sum_{k=1}^{m} b_{k}}, \end{array} $$

where *I*(·) is the indicator function. In contrast, weighted UniFrac, which uses OTU abundance information, is a quantitative measure [19]:

$$\begin{array}{*{20}l} d_{ij}^{W} = \frac{\sum_{k=1}^{m} b_{k} |p_{ik} -p_{jk}|}{\sum_{k=1}^{m} b_{k} |p_{ik} + p_{jk}|}. \end{array} $$

Our basic observation is that phylogenetic distance metrics, which account for the relationship among taxa via a phylogenetic tree, measure the distance among samples using all the variables (i.e., taxa) without variable selection or variable weighting. Since the dimension of microbial data is usually high, much larger than the number of samples, many taxa may provide only weak or no signals. Using a phylogenetic distance without variable weighting or variable selection may or may not be powerful. Instead, corresponding to the unweighted and weighted UniFrac distances, for each sample *i* and taxon *k*, we define the corresponding generalized taxon proportions as

$$\begin{array}{*{20}l} Q_{ik}^{u} = b_{k} I(p_{ik} >0), \qquad Q_{ik}^{w} = b_{k}p_{ik}, \end{array} $$

respectively. Note that the raw weighted UniFrac distance [19] between two samples is exactly the same as the *L*_1_ distance of the weighted generalized taxon proportion between the two samples.

Inspired by a multivariate test for association analysis of rare variants [23], we construct a class of versatile score-based tests such that for a given scenario, at least one of the tests is powerful. Then we combine these tests to maintain high power across a wide range of scenarios. Specifically, for a binary outcome, we use a logistic regression model:

$$\begin{array}{*{20}l} \text{Logit}[\text{Pr}(Y_{i} = 1)] = \beta_{0} +\beta' X_{i} + \sum_{k = 1}^{m}Q_{ik}\varphi_{k}, \end{array} $$

where *Q*_*ik*_ is either $Q_{ik}^{u}$ or $Q_{ik}^{w}$.

For a continuous outcome, we use a linear model:

$$\begin{array}{*{20}l} Y_{i} = \beta_{0} +\beta' X_{i} + \sum_{k = 1}^{m}Q_{ik}\varphi_{k} + \epsilon_{i}, \end{array} $$

where *ε*_*i*_ is an error term with mean 0 and variance *σ*^2^.

We are interested in testing the null hypothesis *H*_0_: *φ*=(*φ*_1_,…,*φ*_*m*_)^′^=0. That is, there is no association between any taxa and the outcome of interest under *H*_0_. The score vector *U*=(*U*_1_,…,*U*_*m*_)^′^ for *φ* is [17, 23–25]:

$$\begin{array}{*{20}l} U =& \sum_{i=1}^{n}(Y_{i}-\hat{\mu}_{i,0})Q_{\textit{i}\cdot}, \end{array} $$

where *Q*_*i*·_=(*Q*_*i*1_,*Q*_*i*2_,…,*Q*_*im*_) and $\hat {\mu }_{i,0}$ is the predicted mean of the outcome of interest (*Y*_*i*_) under *H*_0_. Note that a general weighted score-based test can be written as

$$\begin{array}{*{20}l} T_{\mathrm{G}} = w'U = \sum_{k= 1}^{m} w_{k}U_{k}, \end{array} $$

where *w*=(*w*_1_,…,*w*_*m*_)^′^ is a vector of weights for the *m* generalized taxon proportions. Most existing association tests use the score vector *U* to construct a test statistic, because of the closed form of the score vector *U* and because most of the information in the data is contained in *U*. Therefore, we use *U* to construct the weights for the score vector *U*. Under *H*_0_, we have *U*∼*N*(0,Cov(*U*|*H*_0_)) asymptotically, suggesting that a larger |*U*_*k*_| offers stronger evidence to reject *H*_0,*k*_: *φ*_*k*_=0. Specifically, we choose $w=(U_{1}^{\gamma -1},\dots,U_{m}^{\gamma -1})'$ to weight the score vector for the generalized taxon proportions, leading to a MiSPU test:

$$\begin{array}{*{20}l} T_{\text{MiSPU}(\gamma)} = w'U = \sum_{k= 1}^{m} U_{k}^{\gamma}. \end{array} $$

Since *γ*=1 essentially treats all the variables as equally important while association directions of the generalized taxon proportions may vary, *γ*=1 often yields low power and thus is excluded here. Importantly, as *γ* increases, the MiSPU(*γ*) test puts more weight on the larger components of *U* while gradually ignoring the remaining components. As *γ* goes to infinity, we have

$$\begin{array}{*{20}l} T_{\text{MiSPU}(\infty)} \propto ||U||_{\infty} = \max_{k=1}^{m}|U_{k}|. \end{array} $$

We simply define $T_{\text {MiSPU}(\infty)} = \max _{k=1}^{m}|U_{k}|$. Note that the two versions of *Q*_*ik*_, i.e., $Q_{ik}^{w}$ and $Q_{ik}^{u}$, yield weighted MiSPU_w_ and unweighted MiSPU_u_, respectively.

We use a permutation scheme [23] to calculate the *p* value as the following:

1. Fit the null linear or logistic regression model by regressing *Y* on the covariates *X* under *H*_0_ to obtain $\hat {\mu }_{i,0} = E(Y_{i}|H_{0})$ and residuals $r_{i} = Y_{i} -\hat {\mu }_{i,0}$.
2. Permute the residuals *r*={*r*_*i*_|*i*=1,…,*n*} to obtain a permuted set *r*^(*b*)^.
3. Regress *Q* on the covariates *X* to obtain the residuals $\hat {Q}$.
4. Calculate the new score vector based on the permuted residuals as $U^{(b)} = \sum _{i = 1}^{n} \hat {Q}_{\textit {i}\cdot } r_{i}^{(b)}$ and the corresponding null statistic $T_{\text {MiSPU}}^{(b)} = T_{\text {MiSPU}}(U^{(b)})$.
5. Calculate the *p* value as $\left [\sum _{b=1}^{B} I\left (|T_{\text {MiSPU}}^{(b)}| \geq |T_{\text {MiSPU}}| \right)+1\right ]/(B+1)$ after *B* permutations.

It would be desirable to data-adaptively choose the value of *γ* and the version of the generalized taxon proportion since the optimal choice of them depends on the unknown true association patterns. Like the adaptive SPU (aSPU) test [23], we propose an adaptive MiSPU (aMiSPU) test, which combines the *p* values of multiple MiSPU tests with various values of *γ* and two versions of *Q*_*ik*_. Suppose that we have some candidate values of *γ* in *Γ*, e.g., *Γ*={2,3,…,8,*∞*}, as used in our later simulations and real-data analysis. Then, our combining procedure is to take the minimum *p* value:

$$\begin{array}{*{20}l} T_{\text{aMiSPU}_{\mathrm{u}}} &= \min_{\gamma \in \Gamma} P_{\text{MiSPU}_{\mathrm{u}}(\gamma)}, \\ T_{\text{aMiSPU}_{\mathrm{w}}} &= \min_{\gamma \in \Gamma} P_{\text{MiSPU}_{\mathrm{w}}(\gamma)}, \\ T_{\text{aMiSPU}} &= \min \left\{P_{\text{aMiSPU}_{\mathrm{u}}}, P_{\text{aMiSPU}_{\mathrm{w}}}\right\}. \end{array} $$

Note that we take the minimum *p* value of aMiSPU_u_ and aMiSPU_w_ to form the final aMiSPU test. $T_{\text {aMiSPU}_{\mathrm {u}}}\phantom {\dot {i}\!}$, $T_{\text {aMiSPU}_{\mathrm {w}}}\phantom {\dot {i}\!}$, and *T*_aMiSPU_ are no longer a genuine *p* value, but we can use the permutation to estimate its *p* value, using the same set of null statistics used to calculate the *p* values for the MiSPU tests [23].

We comment on the choice of *Γ* and the version of the generalized taxon proportion. Depending on how many taxa are truly associated with the outcome of interest, one may use a smaller or larger *γ*. For example, if more of the taxa are not associated, a larger *γ* would be desirable. In our numerical simulations and real-data analysis, we have found that *Γ*={2,3,…,8,*∞*} often suffices. MiSPU(8) often gives almost the same results as those of MiSPU(*∞*), suggesting there is no need to use other larger *γ*’s. In practice, we suggest using the aMiSPU test, which combines the strengths (and possibly weaknesses) of various MiSPU tests. The aMiSPU test can be regarded as a rigorous means for multiple testing adjustment with the use of several MiSPU tests, while the results of MiSPU tests may shed light on the underlying association patterns. For example, if a MiSPU with the unweighted generalized taxon proportion gives the most significant *p* value, it may indicate the outcome of interest is more likely to be associated with the abundance changes in rare taxa. If some odd *γ*’s yield more significant results than even *γ*’s, then most or all of the large associations are in the same direction.

Although we focus on rRNA sequencing data, the proposed method can be applied to metagenomic whole-genome shotgun sequencing data as well. Via MEGAN [26], DNA reads (or contigs) can be summarized as OTUs and their counts. Using a standard algorithm, species-specific sequences are assigned to OTUs or taxa near the leaves of a phylogenetic tree, whereas widely conserved sequences are assigned to taxa closer to the root [26]. Once we have OTU abundance data and a phylogenetic tree, aMiSPU can be applied as before.

### Taxon selection

A limitation of most multivariate tests is their inability to select variables: even if the null hypothesis is rejected, they may not give any information on which taxa are (or are not) likely to be associated with the outcome of interest. We note that the aMiSPU test can be used to rank the importance of the taxa. First, if $P_{\text {aMiSPU}_{\mathrm {u}}} < P_{\text {aMiSPU}_{\mathrm {w}}}\phantom {\dot {i}\!}$, we use the unweighted generalized taxon proportion in the subsequent analysis; otherwise, we use the weighted one. For ease of exposition, suppose we choose the weighted one. Second, we estimate the optimal value of $\hat {\gamma } = \text {argmin}_{\gamma \in \Gamma } P_{\text {MiSPU}_{\mathrm {w}}(\gamma)}$ chosen by the aMiSPU_w_ test. If $\hat {\gamma } = \infty $, we can easily find the most significant taxon. Third, suppose $\hat {\gamma } < \infty $, then we assess the relative contribution of each taxon *r* to the aMiSPU_w_ test as $\mathcal {C}_{r} = |U_{r}|^{\hat {\gamma }} / \sum _{j=1}^{m}|U_{r}|^{\hat {\gamma }}$. Fourth, we rank the taxa based on their $\mathcal {C}_{r}$ values, and we can select a few top *k*_1_ taxa, such as *k*_1_=1, or such that the sum of their relative contributions $\sum _{r = 1}^{k_{1}}\mathcal {C}_{r} \geq \alpha _{1}$ with *α*_1_=0.7, say. The choice of *k*_1_ or *α*_1_ determines the trade-off between increasing true positives and increasing false positives.

### The MiSPU package and implementation

We implemented the MiSPU and aMiSPU tests in an R statistical software package called MiSPU, in which a C++ version of UniFrac distances faster than the GUniFrac R package is also provided. The package is available on GitHub (https://github.com/ChongWu-Biostat/MiSPU) and CRAN. We applied MiRKAT from the MIRKAT R package developed by Ni Zhao and Michael Wu at website http://research.fhcrc.org/wu/en/software.html. The SPU and aSPU tests are available in the R package aSPU on CRAN.

### Simulation settings

We used a phylogenetic tree of OTUs from a real throat microbiome data set [27], which consists of 856 OTUs after discarding singleton OTUs. The simulation settings were similar to that used in [16]. Specifically, we generated the OTU counts for each individual via the following steps:

1. Based on a real throat microbiome data set [27], the estimated OTU proportions $(\hat {\pi }_{1},\hat {\pi }_{2},\dots,\hat {\pi }_{856})$ as well as the estimated overdispersion parameter $\hat {\theta }$ were obtained via maximum likelihood.
2. For sample *i*, the observed OTU proportions were randomly generated from a Dirichlet distribution: $(p_{1i},p_{2i},\dots,p_{856i}) \sim \text {Dirichlet}(\hat {\pi }_{1},\hat {\pi }_{2},\dots,\hat {\pi }_{856},\hat {\theta })$.
3. The total count of OTUs for sample *i*, say *n*_*i*_, was randomly drawn from a negative binomial distribution with mean 1000 and size 25. This step mimicked varying total reads per sample.
4. For sample *i*, the observed OTU counts were randomly generated from a multinomial distribution: (*Z*_*i*1_,*Z*_*i*2_,…,*Z*_*i*856_) ∼ Multinomial(*n*_*i*_;*p*_1*i*_,*p*_2*i*_,…,*p*_856*i*_).

The procedure for generating simulated data is available as a function in R package MiSPU. We considered several simulation scenarios that differed in how some OTUs were related to the outcome of interest.

Under simulation scenario 1, we partitioned the 856 OTUs into 20 clusters (lineages) by partitioning around medoids based on the cophenetic distance matrix. The abundance of these 20 OTU clusters varied tremendously, such that each OTU cluster corresponded to some possible bacterial taxa. We assumed that the outcome of interest depended on the abundance cluster that constituted 6.7 % of the total OTU reads. Then we simulated dichotomous outcomes as follows:

$${\small{\begin{aligned} {}\text{Logit}\left(E(Y_{i}|X_{i},Z_{i})\right) = 0.5 \, \text{scale}(X_{1i} + X_{2i}) + \beta \, \text{scale} \left(\sum_{j\in A} Z_{ij}\right), \end{aligned}}} $$

where *β* was the effect size and scale(*Z*_*i*1_) standardized the sample mean of *Z*_*i*·_’s to 0 and the standard deviation to 1. For continuous outcomes, we simulated under the model

$$\begin{array}{*{20}l} Y_{i}= 0.5 \, \text{scale}(X_{1i} + X_{2i}) + \beta \, \text{scale} \left(\sum_{j\in A} Z_{ij}\right) + \epsilon_{i}, \end{array} $$

where *ε*_*i*_∼*N*(0,1). *X*_1*i*_ and *X*_2*i*_ were the covariates to be adjusted for, and *A* was the index set of the selected OTU cluster. *X*_1*i*_ was generated from a Bernoulli distribution Bin(1,0.5), while *X*_2*i*_ was from a standard normal distribution *N*(0,1). To consider the effect of potential confounders, we studied the case where *X*_2*i*_ and *Z*_*i*_ were correlated, specifically, $X_{2i} = \text {scale} \left (\sum _{j\in A} Z_{ij}\right) + N(0,1)$. We varied the effect size *β* to mimic different magnitudes of association.

Under simulation scenario 2, we partitioned all the OTUs into 40 clusters and assumed the outcome was associated with the abundance cluster with only three OTUs. Under simulation scenarios 3, 4, and 5, we assumed that the outcome of interest was associated with the abundance cluster with 24.8 %, 16.6 %, and 1.5 % of the total OTU reads, respectively. Under simulation scenario 6, we assumed the outcome was associated with 50 randomly selected OTUs.

For all the simulation scenarios, we considered using MiSPU_u_ and MiSPU_w_ with *γ*=2,3,…,8. We combined the MiSPU tests to get aMiSPU_u_, aMiSPU_w_, and aMiSPU. We compared aMiSPU with MiRKAT with the weighted and unweighted UniFrac kernels (*K*_w_ and *K*_u_, respectively), the Bray–Curtis kernel (*K*_BC_), and a generalized UniFrac kernel with *α*=0.5 (*K*_5_). Additionally, we also applied the optimal MiRKAT, which combines the above four kernels.

Throughout the simulations, the sample size and test significance level were fixed at 100 and *α*=0.05, respectively. The results were based on 1000 independent replicates for *β*≠0 and 10,000 independent simulations for *β*=0.

## Results

In this section, we present the simulation results from MiSPU and MiRKAT, as well as the results for three real-data sets. We used several published and de-identified human datasets without any confidential information, thus, accordingly there was no human subject involvement and no need for an IRB approval.

### Numerical simulation results for type I error and power

To save space, we focus on a few simulation set-ups with a binary outcome. The extensive simulation results with different association patterns and outcomes were similar to those presented below and, thus, are relegated to the supporting information in Additional file 1.

First, the type I error rates of MiSPU and aMiSPU across different simulation set-ups were satisfactorily controlled when the confounders were suitably adjusted (Table 1). When the covariates were independent of the microbiome composition, MiSPU and aMiSPU controlled type I error rate well no matter whether we adjusted for *X* or not. In comparison, when *X* and *Z* were correlated, adjusting for *X* was necessary: failing to adjust for *X* led to an inflated type I error. To save space, we only show some results. The type I errors at the nominal *α*=0.01 level were also investigated with the same conclusion (Additional file 1: Table S1).

**Table 1:** Empirical type I error rates for MiSPU and aMiSPU for scenario 1 with a binary outcome

	MiSPU_w_(2)	MiSPU_w_(*∞*)	aMiSPU_w_	aMiSPU_u_	aMiSPU
*X* ⊥ ⊥*Z*, adjust *X*	0.052	0.050	0.052	0.049	0.048
*X* ⊥ ⊥*Z*, no adjust *X*	0.051	0.051	0.052	0.049	0.049
*X*∼*Z*, adjust *X*	0.043	0.038	0.043	0.049	0.040
*X*∼*Z*, no adjust *X*	0.091 ^a^	0.119 ^a^	0.112 ^a^	0.053	0.088 ^a^

The type I error rate was evaluated for situations in which the covariates were independent of the OTUs (*X* ⊥ ⊥*Z*) or correlated with the OTUs (*X*∼*Z*) based on 10,000 simulated data sets at *α*=0.05

^a^Inflated type I error rates

**Fig. 2:**
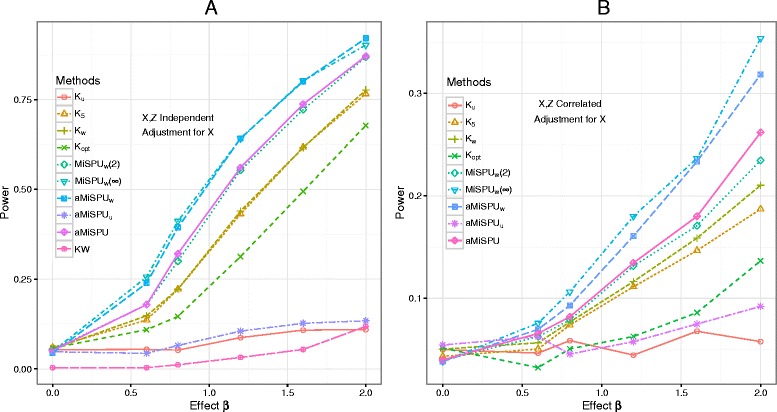
Type I error and power comparison for scenario 1 with a binary outcome. A selected phylogenetic cluster (6.7 %) of the OTUs was associated with the outcome. **a** *X* and *Z* are independent and **b** *X* and *Z* are correlated. *K*_u_, *K*_w_, and *K*_5_ represent MiRKAT results from the unweighted UniFrac kernel, weighted UniFrac kernel, and generalized UniFrac kernel with *α*=0.5, respectively. *K*_opt_ represents the simulation results for optimal MiRKAT considering the Bray–Curtis kernel, unweighted UniFrac kernel, weighted UniFrac kernel, and generalized UniFrac kernel. MiSPU_w_(2), MiSPU_w_(*∞*), and aMiSPU_w_ represent the MiSPU_w_ test with *γ*=2,*∞* and aMiSPU_w_ summarizing *γ*=2,3,…,8,*∞*, respectively. aMiSPU_u_ and aMiSPU represent the test summarizing *γ*=2,3,…,8,*∞* with unweighted generalized taxon proportion and combining aMiSPU_u_ and aMiSPU_w_, respectively. KW represents Kruskal-Wallis test. Results were presented at *n*=100. *KW* Kruskal–Wallis test

Figure 2 shows statistical power with a binary outcome in simulation scenario 1, in which a phylogenetic cluster with 6.7 % OTUs was associated with the outcome. For all the tests considered, the power increased when the effect size increased. Due to the upweighting of the microbial taxa more likely to be informative, a MiSPU_w_ test was much more powerful than a MiRKAT test, regardless of whether *X* and *Z* were correlated or not. Because only a few taxa were related to the outcome of interest, a MiSPU(*γ*) test with a larger *γ* performed slightly better than that with a smaller *γ*. Nevertheless, MiSPU_w_(2) still performed much better than any MiRKAT. Compared to MiSPU_w_(*∞*), aMiSPU_w_ combining different weights with various *γ* values lost some power but still maintained power considerably higher than that of many other tests. As expected, by ignoring the phylogenetic information of the microbiome data, the SPU and the aSPU tests [23] failed to achieve high power (not shown). Since there were some abundant OTUs in the informative cluster *A*, unweighted UniFrac suffered from a loss of power and led to the failure of aMiSPU_u_ to improve power. However, aMiSPU combining aMiSPU_u_ and aMiSPU_w_ lost only little power compared to aMiSPU_w_. Note that when *X* and *Z* were independent, adjusting for the covariates *X* or not had a minimum effect on the power (Additional file 1: Figure S1). The simulation results for continuous outcomes were similar (Additional file 1: Figures S2 and S3).

**Fig. 3:**
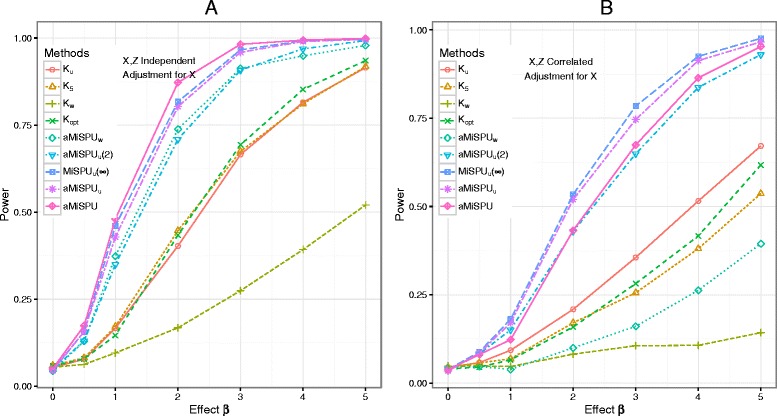
Type I error and power comparison for scenario 2 with a binary outcome. A selected phylogenetic cluster (0.35 %) of the OTUs was associated with the outcome. *X* and *Z* are independent (**a**) or correlated (**b**). *K*_u_, *K*_w_, and *K*_5_ represent MiRKAT results from the unweighted UniFrac kernel, weighted UniFrac kernel, and generalized UniFrac kernels with *α*=0.5, respectively. *K*_opt_ represents the simulation results for optimal MiRKAT considering the Bray–Curtis kernel, unweighted UniFrac kernel, weighted UniFrac kernel, and generalized UniFrac kernel. MiSPU_u_(2), MiSPU_u_(*∞*), and aMiSPU_u_ represent the MiSPU_u_ test with *γ*=2,*∞* and aMiSPU_u_ summarizing *γ*=2,3,…,8,*∞*, respectively. aMiSPU_w_ and aMiSPU represent the test summarizing *γ*=2,3,…,8,*∞* with weighted generalized taxon proportion and combining aMiSPU_u_ and aMiSPU_w_, respectively. Results were presented at *n*=100

Figure 3 shows the statistical power with a binary outcome in simulation scenario 2, where a small phylogenetic cluster that contains only three OTUs was associated with the outcome. We again show the empirical power curves when *X* and *Z* were independent (Fig. 3a) and when *X* and *Z* were correlated (Fig. 3b). The results are similar to those of simulation scenario 1, except that aMiSPU_u_ performed better than aMiSPU_w_. aMiSPU, which combines aMiSPU_u_ and aMiSPU_w_, lost only little power compared to the best choice MiSPU, but remained much more powerful than any of MiRKAT. As expected, the weighted UniFrac kernel was the least powerful.

Other simulations showed consistently that aMiSPU generally outperformed MiRKAT and aSPU when a phylogenetic cluster was associated with the outcome (Additional file 1: Figures S4, S5, and S6). However, when some randomly selected OTUs were associated with the outcome (scenario 6), the aSPU test was the winner (Additional file 1: Figure S7); however, we comment that this scenario may not be realistic.

In practice, the true state of nature can vary from case to case. The simulation results show that the power of MiRKAT essentially depends on the chosen kernel; a poor choice of the kernel leads to a tremendous loss of power. In contrast, MiSPU uses the generalized taxon proportion *Q*_*ik*_ and puts higher weight on taxa more likely to be informative, achieving much higher power than MiRKAT in most situations. The performance of MiSPU is also dependent on the choice of *γ* and the version of the generalized taxon proportion: a better choice leads to higher power. However, aMiSPU alleviates this problem by combining MiSPUs with different *γ*’s and the two versions of the generalized taxon proportion, and it is the overall winner over a wide range of different scenarios.

Univariate testing on each OTU or taxon one by one incurs a heavy burden for a correction for multiple testing. Often the easy-to-use but conservative Bonferroni method is applied, leading to reduced power. Compared to multivariate testing methods, such as MiSPU and MiRKAT, the power of the nonparametric Kruskal–Wallis test [28, 29] was very low (Fig. 2a). Even worse, many parametric univariate tests, due to their strong parametric assumptions on the distributions or parametric specifications on the mean-variance forms for the OTU counts, may have inflated false positive rates, as pointed out by others [12, 30]. For example, in our simulations under scenario 1, the empirical type I error rates for DESeq2 [31] and metagenomeSeq-fitZig [32] were inflated. Accordingly, we did not further investigate their power properties. Relevantly and importantly, univariate tests encounter the so-called curse of the compositionality problem: since the increased (or decreased) relative abundance of some OTUs necessarily leads to other (null or unmodified) OTUs having opposite changes in their relative abundance, there are false positives for some null OTUs. In contrast, multivariate joint testing methods, such as PERMANOVA, MiRKAT, and aMiSPU, do not suffer from this curse of the compositionality problem.

### Numerical simulation results for taxon selection

Beyond an overall assessment of association, several methods [28, 29, 31–33] have been developed for identifying specific OTUs driving a detected association. For example, since the compositions of potentially pathogenic bacteria across healthy and disease populations might be different, identifying such bacteria is of interest. One by-product of the aMiSPU test is a ranking of the importance of the taxa. We evaluated taxon selection using simulated data under scenario 1 with an effect size equal to 2, and compared the results to those of the other metagenomic tools, metagenomeSeq-fitZig [32], a Kruskal–Wallis test as used in LEFSe (linear discriminant analysis effect size) [28] and STAMP [29], and DESeq2 [31], a representative for RNA-seq analysis.

The simulation results under scenario 1 are summarized in Table 2. The informative OTU set contained 57 OTUs. On average, the taxon set selected by aMiSPU contained 58.5 OTUs, 27.2 of which were truly informative. In contrast, fitZig [32] selected 157 OTUs and only 12.3 OTUs were truly informative. Perhaps due to the failure to consider the fact that most OTUs in a microbiome association study are rare, DESeq2 and the KW test performed poorly with a too small mean number of true positives. Under scenario 1, we chose a relatively abundant OTU cluster that contained 57 OTUs to be related to the outcome. As expected, incorporating phylogenetic tree information helped us select truly informative abundant OTUs, thus aMiSPU performed better. In contrast, with only a moderate effect size for each informative OTU, a univariate association test was much less powerful in identifying informative OTUs.

**Table 2:** Sample means (standard deviations in parentheses) of the total number of selected OTUs (Total), and of the numbers of true positives and false positives

	Method	Total	TP	FP
*X* ⊥ ⊥*Z*	fitZig	157.0 (49.4)	12.3 (4.7)	144.6 (45.7)
	DESeq2	20.4 (4.8)	3.4 (1.5)	17.0 (4.4)
	KW	25.8 (6.1)	3.5 (1.6)	22.3 (5.7)
	aMiSPU with *α*_1_=.7	234.1 (241.5)	42.6 (18.5)	191.6 (234.8)
	aMiSPU with *k*_1_=1	58.5 (76.9)	27.2 (20.6)	31.3 (78.1)

Based on 1000 simulation replications under scenario 1, by fitZig [32], DESeq2 [31], KW test, aMiSPU with *α*_1_=.7, or aMiSPU with *k*_1_=1. For fitZig, DESeq2 and KW test, cutoff 5×10^−5^, 0.05, 0.05 were chosen, respectively

*FP* number of false positives, *KW* Kruskal–Wallis test, *OTU* operational taxonomic unit, *TP* number of true positives

### Analysis of a gut microbiome data set for gender and diet effects

Diet strongly affects human health, partly by modulating gut microbiome composition. Wu et al. [34] investigated the association of dietary and environmental variables with the gut microbiota, where the diet information was converted into a vector of micro-nutrient intakes. In this cross-sectional study, 98 healthy volunteers were enrolled and habitual long-term diet information was collected using a food frequency questionnaire. The questionnaires were converted to intake amounts of 214 micro-nutrients, which was further normalized via a residual method to standardize for caloric intake. Stool samples were collected, from which DNA samples were analyzed and denoised prior to taxonomic assignment. The denoised sequences were then analyzed by the QIIME pipeline [15] with the default parameter settings, yielding 3071 OTUs after discarding the singleton OTUs.

**Fig. 4:**
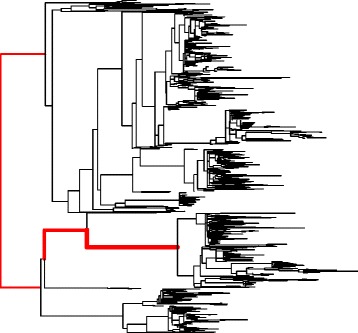
Phylogenetic tree of *Bacteroides* enterotypes for a gut microbiome data set. *Black edges* stand for non-associated signals, while *red edges* stand for the associated signals. The width of the edges stands for the magnitude of the association

Increasing evidence suggests that there is a sex difference in the human gut microbiome, which in turn modulates many pathological and physiological processes [35, 36]. However, no significant sex effect was detected using PERMANOVA based on this data set [34]. We thus re-analyzed the data set for the gender effect by applying MiRKAT and MiSPU with 100,000 permutations. Using MiRKAT, we found the *p* values from weighted UniFrac, unweighted UniFrac, and the Bray–Curtis kernel to be 0.035, 0.039, and 0.087, respectively. The optimal MiRKAT generated a *p* value of 0.080, failing to reject the null hypothesis even at the *α*=0.05 significance level. In comparison, MiSPU_w_(2), MiSPU_w_(3), MiSPU_w_(8), and MiSPU_w_(*∞*) provided *p* values of 0.011, 0.0018, 0.0022, and 0.0022, respectively. MiSPU_w_(3) provided the most significant *p* value, suggesting that there is a sparse association pattern between gut microbiome composition and gender status, and the large associations between gender and and one or few microbial taxa were in the same direction. aMiSPU, combining the weighted and unweighted generalized taxon proportions and *γ*={2,3,…,8,*∞*}, yielded a *p* value of 0.0058, rejecting the null hypothesis at the *α*=0.01 significance level, suggesting an association between gender status and microbiome composition. Note that perhaps due to the relatively high signal sparsity, previous studies [34, 37] using distance-based methods [13] failed to find any association. Unlike MiRKAT and distance-based analyses, the aMiSPU test can be used for taxon selection. Since MiSPU_w_(3) provided the most significant *p* value, we used the weighted generalized taxon proportion and $\hat {\gamma } = 3$. We found that a taxon in *Bacteroides* explained more than 90 % of the relative contributions. The top four taxa all came from *Bacteroides*, suggesting that gender was likely associated with *Bacteroides*, but independent of other enterotypes (Fig. 4).

**Fig. 5:**
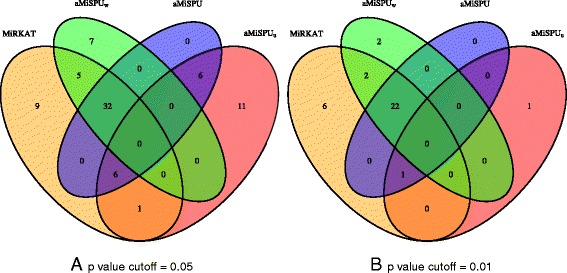
Venn diagram of detected associations for the gut microbiome data set. In the testing, 214 nutrients are included. Results are shown for a *p* value cutoff of 0.05 (**a**) and 0.01 (**b**). MiRKAT represents the results for optimal MiRKAT considering the Bray–Curtis kernel, unweighted UniFrac kernel, weighted UniFrac kernel, and generalized UniFrac kernel. aMiSPU_w_ represents a test combining MiSPU_w_ with *γ*=2,*∞*. aMiSPU_u_ and aMiSPU represent the test summarizing *γ*=2,3,…,8,*∞* and combining aMiSPU_u_ and aMiSPU_w_, respectively

One goal of the study is to identify nutrients that are associated with the gut microbiome composition. We re-analyzed the data from the gut samples by using MiRKAT [16] and aMiSPU. Specifically, we applied the optimal MiKRAT test to analyze the association between each nutrient and microbial community composition by combining the weighted and unweighted UniFrac distances, generalized UniFrace distance with *α*=0.5, and the Bray–Curtis distance (after being transformed to the corresponding similarity matrices). We further applied aMiSPU_u_ and aMiSPU_w_ with *γ*=2,3,…,8,*∞*. Then we combined aMiSPU_u_ and aMiSPU_w_ for aMiSPU. Figure 5 shows that there was no uniformly most powerful test. Depending on the unknown truth, including specific association directions and effect sizes, a given test may or may not be the most powerful. Perhaps due to the sparse association between some of the nutrients and microbial community composition, aMiSPU_u_ detected some signals undiscovered by others.

### Analysis of a gut microbiome data set for association with inflammatory bowel disease

The disruption of the gut microbiota is thought to have an important effect on the etiology of IBDs such as Crohn’s disease (CD) and ulcerative colitis (UC). Willing et al. [6] explored the composition of the IBD gut microbiome and identified some IBD-associated bacterial signatures. In this cohort study, 40 twin pairs who were concordant or discordant for CD or UC were collected and the compositions of microbial communities in feces samples were determined via 454 pyrotag sequencing. Sequences were checked for quality and those that were less than 200 base pairs in length, contained incorrect primer sequences, or contained more than one ambiguous base were discarded [6].

We tested the association between disease status and the overall microbiome composition via MiRKAT and MiSPU using 10,000 permutations. MiRKAT yielded *p* values from weighted UniFrac, unweighted UniFrac, and Bray–Curtis kernels of 0.223, 0.059, and 0.475, respectively. The optimal MiRKAT generated a *p* value of 0.144, failing to reject the null hypothesis even at the *α*=0.10 significance level. In comparison, MiSPU_u_(2), MiSPU_u_(3), and MiSPU_u_(*∞*) provided *p* values of 0.036, 0.053, and 0.084, respectively. The aMiSPU test, combining the weighted and unweighted generalized taxon proportions and *γ*∈{2,3,…,8,*∞*}, yielded a *p* value of 0.097, slightly smaller than 0.10, rejecting the null hypothesis at the 0.10 significance level. None of these tests could reject the null hypothesis at the *α*=0.05 significance level, perhaps due to the small sample size. Note that, perhaps because disease status was more likely to be associated with abundance changes in rare taxa, MiSPU_u_ provided a more significant *p* value than MiSPU_w_.

### Analysis of a throat microbiome data set for smoking effects

Cigarette smokers have an increased risk of infection involving the respiratory tract. Recently, a microbiome-profiling study was conducted to investigate the effect of smoking on the oropharyngeal and nasopharyngeal bacterial communities [27]. In brief, they analyzed bacterial colonization in the upper airway in 29 healthy cigarette smokers compared with 33 non-smokers. For each DNA sample, 102 of the bacterial rRNA genes were PCR-amplified using individually barcoded primer sets. Then pyrosequences were denoised prior to taxonomic assignment [38]. Using the QIIME pipeline [15], sequences were clustered at 97 % similarity level into OTUs. They excluded the samples with fewer than 500 reads and OTUs with only one read, leading to 60 samples remaining and 856 OTUs. Gender (*p*<0.05) and antibiotic use within the last 3 months were collected.

In a previous analysis [16], MiKRAT was applied to test the association between smoking and microbial community composition while adjusting for the effect of gender and antibiotic status. Using MiRKAT, we found the *p* values from weighted UniFrac, unweighted UniFrac, and Bray–Curtis kernels to be 0.0048, 0.014, and 0.002, respectively. The optimal MiRKAT generated a *p* value of 0.0031 [16]. In comparison, MiSPU_w_(2), MiSPU_w_(7), MiSPU_w_(8), and MiSPU_w_(*∞*) yielded *p* values of 0.0147, 0.0011, 0.0013, and 0.0012, respectively. MiSPU(8) and MiSPU(*∞*) provided almost the same *p* values, further confirming that there was no need to use other larger *γ*’s. MiSPU_w_(7) provided the most significant *p* value, suggesting that there was a sparse association pattern and the large associations between smoking status and one or few microbial taxa were in the same direction. aMiSPU_w_, combining all the MiSPU_w_ tests with *γ*=2,3,…,8,*∞*, yielded a *p* value of 0.0029. aMiSPU_u_, combining all the MiSPU_u_ tests with *γ*=2,3,…,8,*∞*, yielded a *p* value of 0.0431, less significant than that from aMiSPU_w_ and suggesting that some abundant taxa may be correlated with smoking status. The aMiSPU test, combining aMiSPU_w_ and aMiSPU_u_, yielded a *p* value of 0.0050, confirming the results of the previous analysis, though it was slightly larger than that of the optimal MiRKAT.

## Discussion

We have proposed and studied a class of MiSPU tests and an adaptive version (aMiSPU) for an overall association between a microbial community and an outcome of interest. The aMiSPU test is based on the score vector for a new variable called generalized taxon proportion, which combines taxon abundance information with phylogenetic tree information, rendering it both computationally efficient and general to cover a wide range of applications with binary or quantitative outcomes and possible covariates. Our major contribution is that, by recognizing the limitation of the existing methods without variable selection or variable weighting, we propose the use of the two versions of the generalized taxon proportion to account simultaneously for the effects of relative abundances of microbial taxa and that of branch lengths in a phylogenetic tree, and apply many possible weights indexed by a single parameter *γ*≥2 to weight the taxa differentially. This approach can maintain high power in a wide range of scenarios.

Besides assessing the overall association with a microbial community, one may be interested in finding possible taxa driving a detected association. Unlike MiRKAT [16] and other distance-based methods [13, 20, 39], which are unable for taxon selection, the proposed aMiSPU test can be used to rank the importance of taxa and thus, provide some insights on which taxa are likely to be associated with the outcome of interest.

A few modifications or extensions are possible. First, in our current implementation of MiSPU, we propose the use of a generalized taxon proportion and weight it based on its corresponding score component; we may explicitly consider some interactions among the taxa. Second, we take the minimum *p* value to combine the results of multiple MiSPU tests. Instead, we may apply other methods that may perform better in some scenarios [40]. Finally, though we focused on a binary and continuous outcome of interest, it might be of interest and possible to extend MiSPU to cases with a multivariate, longitudinal or survival outcome in a general framework of regression.

## Conclusions

We have evaluated the MiSPU and aMiSPU tests extensively using both simulated and real data, revealing their excellent performance across many situations. As noted, aMiSPU maintains high power across a wide range of scenarios, though the identity of the most powerful MiSPU test is expected to change with the varying scenarios. In comparison with other multivariate joint tests, we found that aMiSPU was often much more powerful, and thus we recommend its use in practice. An R package MiSPU implementing the aMiSPU test and a C++ version of the UniFrac distance calculation are available on GitHub (https://github.com/ChongWu-Biostat/MiSPU) and CRAN.

## Additional file

Additional file 1 Seven supporting figures and one supporting table. A description of each is given within the file. (PDF 3778 kb)

## Abbreviations

aMiSPU: adaptive microbiome-based sum of powered score
aSPU: adaptive sum of powered score
CD: Crohn’s disease
IBD: inflammatory bowel disease
MiRKAT: microbiome regression-based kernel association test
MiSPU: microbiome-based sum of powered score
OTU: operational taxonomic unit
SPU: sum of powered score
UC: ulcerative colitis
