# Supplementary material for: An adaptive association test for microbiome data
Source: Genome Med. 2016 May 19;8:56. doi: 10.1186/s13073-016-0302-3 (PMC4872356; doi:10.1186/s13073-016-0302-3)
Supplement: Additional file 1 — Seven supporting figures and one supporting table. A description of each is given within the file. (PDF 3778 kb) [file 13073_2016_302_MOESM1_ESM.pdf]

Additional file 1
Supplementary tables and figures

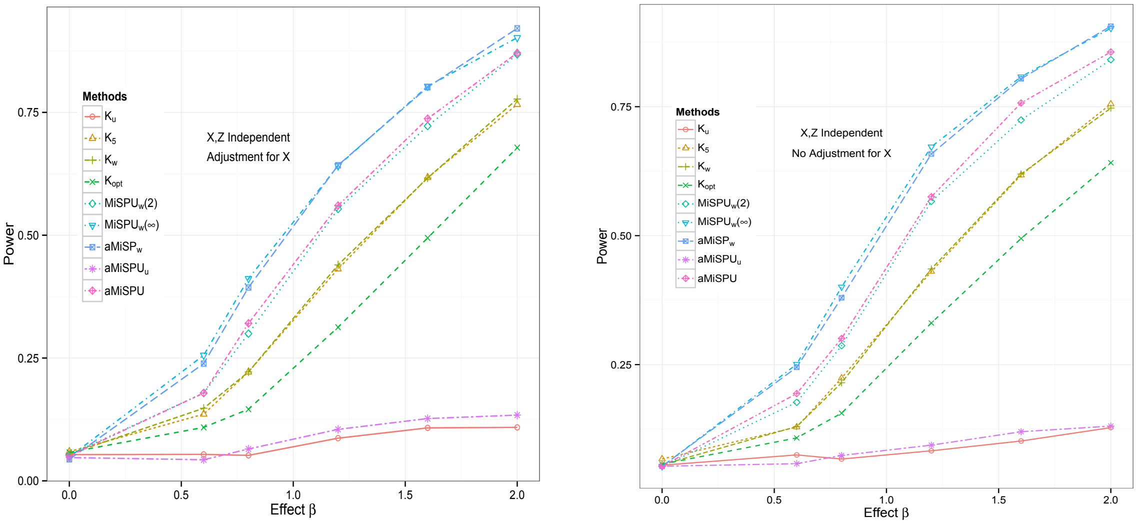

(a) $X \perp\!\!\!\perp Z$, adjustment for X (b) $X \perp\!\!\!\perp Z$, no adjustment for X

Figure S1: **Type I error and power comparison for scenario 1 with binary outcome when X and Z were independent.** A selected phylogenetic cluster (**6.7%**) of the OTUs were associated with the outcome. Results were shown for did (a) or did not (b) adjust for covariates X. K_u, K_5 and K_w represent MiRKAT results from the unweighted UniFrac kernel, unweighted UniFrac kernel and generalized UniFrac kernels with $\alpha = 0.5$, respectively. K_{opt} represents the simulation results for optimal MiRKAT combining Bray-Curtis kernel, unweighted UniFrac kernel, weighted UniFrac kernel and generalized UniFrac kernel. MiSPU$_w(2)$, MiSPU$_w(\infty)$ and aMiSPU$_w$ represent MiSPU$_w$ test with $\gamma = 2, \infty$ and aMiSPU$_w$ summarizing $\gamma = 2, 3, \ldots, 8, \infty$, respectively. aMiSPU$_u$ and aMiSPU represent the test summarizing $\gamma = 2, 3, \ldots, 8, \infty$ and combing aMiSPU$_u$ and aMiSPU$_w$, respectively. Results were presented at $n = 100$.

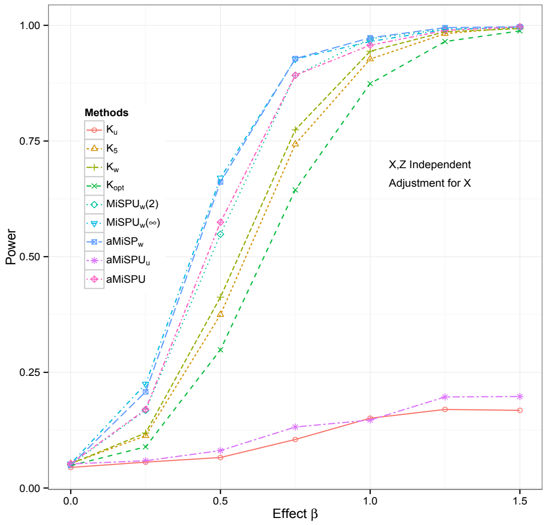

(a) $X \perp\!\!\!\perp Z$, adjustment for X

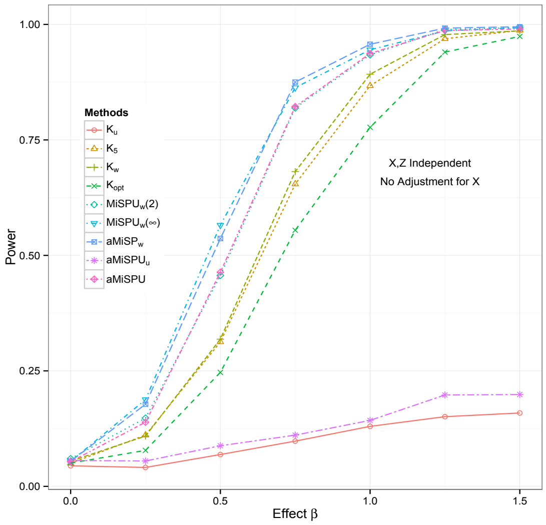

(b) $X \perp\!\!\!\perp Z$, no adjustment for X

Figure S2: **Type I error and power comparison for scenario 1 with continuous outcome when X and Z were independent.** A selected phylogenetic cluster (**6.7%**) of the OTUs were associated with the outcome. Results were shown for did (a) or did not (b) adjust for covariates X. K_u, K_5 and K_w represent MiRKAT results from the unweighted UniFrac kernel, unweighted UniFrac kernel and generalized UniFrac kernels with $\alpha = 0.5$, respectively. K_{opt} represents the simulation results for optimal MiRKAT combining Bray-Curtis kernel, unweighted UniFrac kernel, weighted UniFrac kernel and generalized UniFrac kernel. $\text{MiSPU}_w(2)$, $\text{MiSPU}_w(\infty)$ and aMiSPU_w represent MiSPU_w test with $\gamma = 2, \infty$ and aMiSPU_w summarizing $\gamma = 2, 3, \ldots, 8, \infty$, respectively. aMiSPU_u and aMiSPU represent the test summarizing $\gamma = 2, 3, \ldots, 8, \infty$ and combing aMiSPU_u and aMiSPU_w, respectively. Results were presented at $n = 100$.

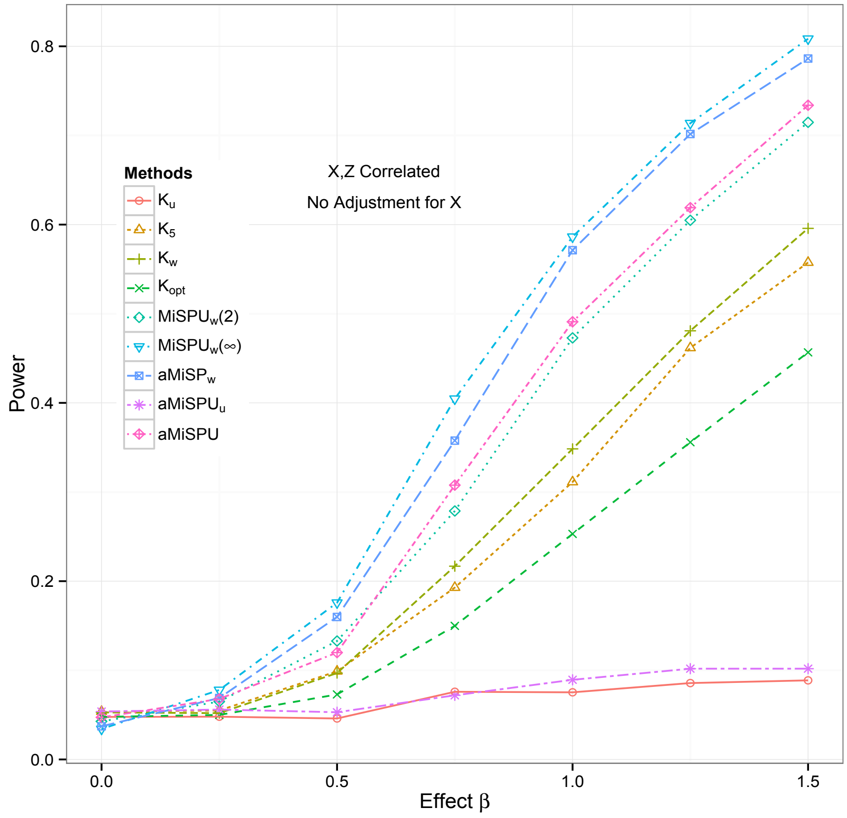

Figure S3: **Type I error and power comparison for scenario 1 with continuous outcome when X and Z were correlated.** A selected phylogenetic cluster (**6.7%**) of the OTUs were associated with the outcome, and covariates (X) and microbiome composition (Z) were correlated such that $X_{2i} = \text{scale}(\sum_{j \in \mathcal{A}} Z_{ij}) + N(0,1)$ where $\mathcal{A}$ is the selected cluster. K_u, K_5 and K_w represent MiRKAT results from the unweighted UniFrac kernel, unweighted UniFrac kernel and generalized UniFrac kernels with $\alpha = 0.5$, respectively. K_{opt} represents the simulation results for optimal MiRKAT combining Bray-Curtis kernel, unweighted UniFrac kernel, weighted UniFrac kernel and generalized UniFrac kernel. $\text{MiSPU}_w(2)$, $\text{MiSPU}_w(\infty)$ and aMiSPU_w represent MiSPU_w test with $\gamma = 2, \infty$ and aMiSPU_w summarizing $\gamma = 2, 3, \ldots, 8, \infty$, respectively. aMiSPU_u and aMiSPU represent the test summarizing $\gamma = 2, 3, \ldots, 8, \infty$ and combing aMiSPU_u and aMiSPU_w, respectively. Results were presented at $n = 100$.

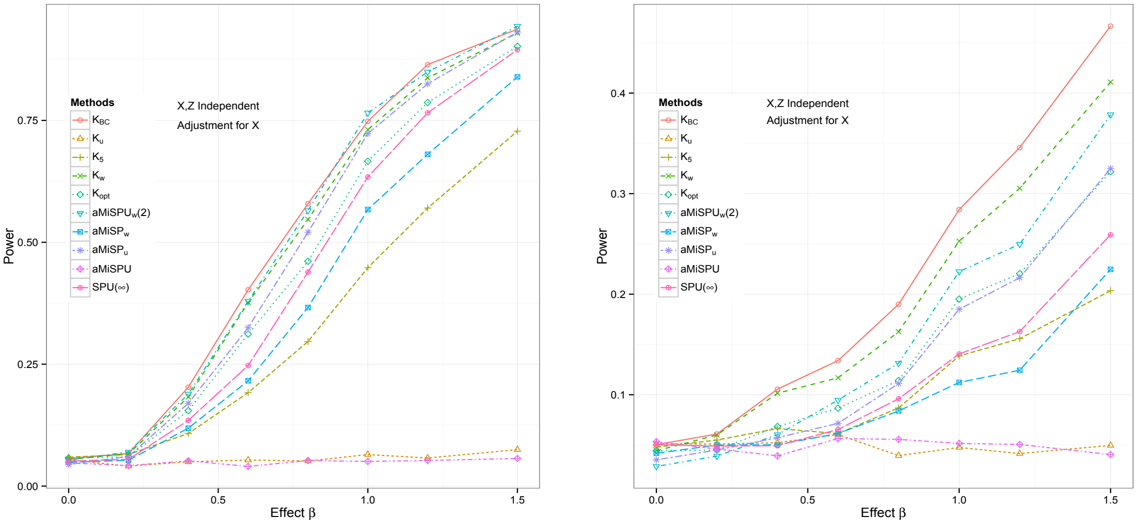

(a) $X \perp\!\!\!\perp Z$ (b) $X \sim Z$

Figure S4: **Type I error and power comparison for scenario 3 with binary outcome when adjusting for covariates.** A selected phylogenetic cluster (**24.8%**) of the OTUs were associated with the outcome. Results were shown for X and Z were independent (a) or correlated (b). K_{BC}, K_u, K_5 and K_w represent MiRKAT results from the Bray-Curtis kernel, unweighted UniFrac kernel, unweighted UniFrac kernel and generalized UniFrac kernels with $\alpha = 0.5$, respectively. K_{opt} represents the simulation results for optimal MiRKAT combining Bray-Curtis kernel, unweighted UniFrac kernel, weighted UniFrac kernel and generalized UniFrac kernel. MiSPU$_w$(2) and aMiSPU$_w$ represent MiSPU$_w$ test with $\gamma = 2$ and aMiSPU$_w$ summarizing $\gamma = 2, 3, \ldots, 8, \infty$, respectively. aMiSPU$_u$ and aMiSPU represent the test summarizing $\gamma = 2, 3, \ldots, 8, \infty$ and combing aMiSPU$_u$ and aMiSPU$_w$, respectively. $SPU_(\infty)$ represents the SPU tests with OTUs count and $\gamma = \infty$. Results were presented at $n = 100$.

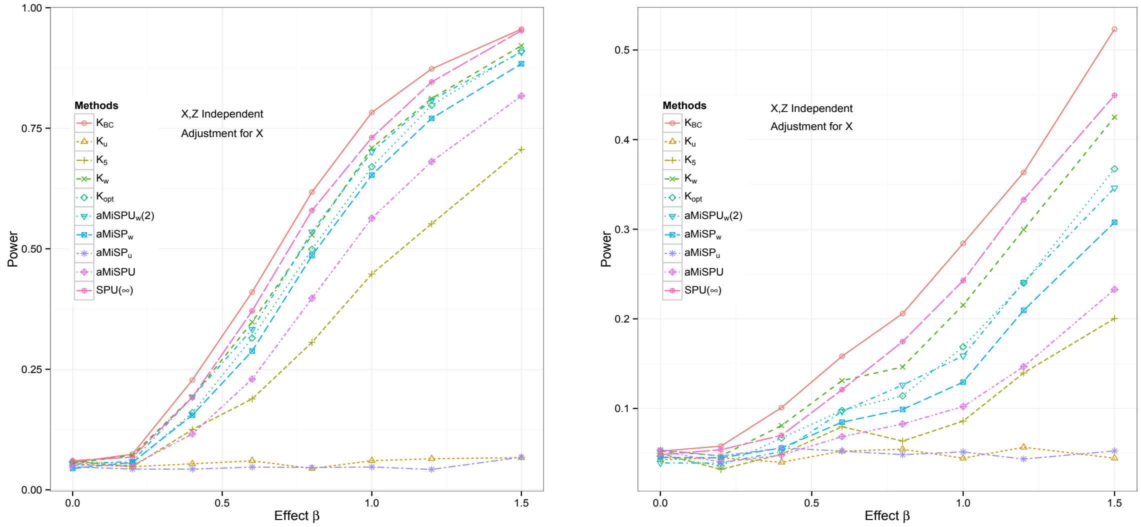

(a) $X \perp\!\!\!\perp Z$ (b) $X \sim Z$

Figure S5: **Type I error and power comparison for scenario 4 with binary outcome while adjusting for covariates.** A selected phylogenetic cluster (**16.6%**) of the OTUs were associated with the outcome. Results were shown for X and Z were independent (a) or correlated (b). K_{BC}, K_u, K_5 and K_w represent MiRKAT results from the Bray-Curtis kernel, unweighted UniFrac kernel, unweighted UniFrac kernel and generalized UniFrac kernels with $\alpha = 0.5$, respectively. K_{opt} represents the simulation results for optimal MiRKAT combining Bray-Curtis kernel, unweighted UniFrac kernel, weighted UniFrac kernel and generalized UniFrac kernel. MiSPU$_w$(2) and aMiSPU$_w$ represent MiSPU$_w$ test with $\gamma = 2$ and aMiSPU$_w$ summarizing $\gamma = 2, 3, \ldots, 8, \infty$, respectively. aMiSPU$_u$ and aMiSPU represent the test summarizing $\gamma = 2, 3, \ldots, 8, \infty$ and combing aMiSPU$_u$ and aMiSPU$_w$, respectively. $SPU(\infty)$ represents the SPU tests with the OTUs count and $\gamma = \infty$. Results were presented at $n = 100$.

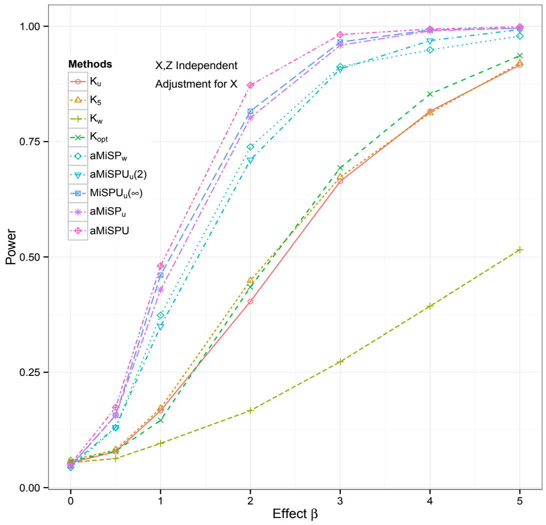

(a) $X \perp\!\!\!\perp Z$

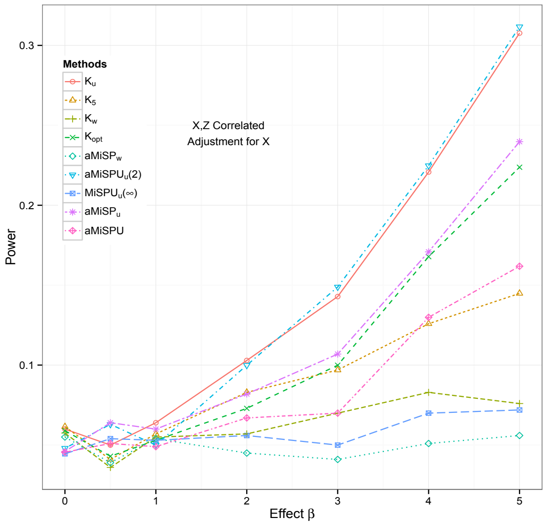

(b) $X \sim Z$

Figure S6: **Type I error and power comparison for scenario 5 with binary outcome when adjusting for covariates.** A selected phylogenetic cluster (**1.5%**) of the OTUs were associated with the outcome. Results were shown for X and Z were independent (a) or correlated (b). K_u, K_5 and K_w represent MiRKAT results from the unweighted UniFrac kernel, unweighted UniFrac kernel and generalized UniFrac kernels with $\alpha = 0.5$, respectively. K_{opt} represents the simulation results for optimal MiRKAT combining Bray-Curtis kernel, unweighted UniFrac kernel, weighted UniFrac kernel and generalized UniFrac kernel. MiSPU$_u(2)$, MiSPU$_u(\infty)$ and aMiSPU$_u$ represent MiSPU$_u$ test with $\gamma = 2, \infty$ and aMiSPU$_u$ summarizing $\gamma = 2, 3, \ldots, 8, \infty$, respectively. aMiSPU$_w$ and aMiSPU represent the test summarizing $\gamma = 2, 3, \ldots, 8, \infty$ and combing aMiSPU$_u$ and aMiSPU$_w$, respectively. Results were presented at $n = 100$.

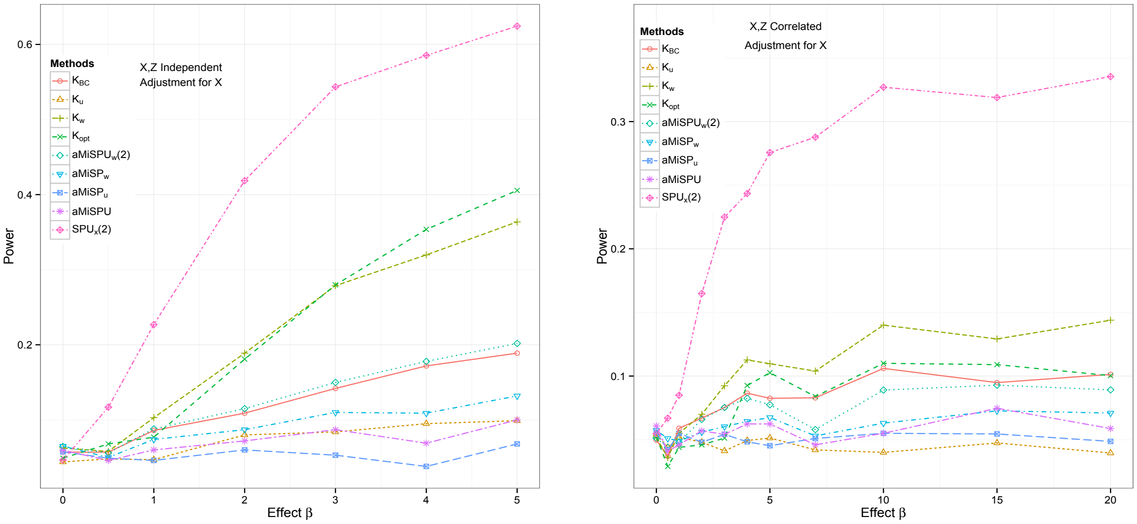

(a) $X \perp\!\!\!\perp Z$ (b) $X \sim Z$

Figure S7: **Type I error and power comparison for scenario 6 with binary outcome while adjusting for covariates.** A randomly selected OTUs (**5.8%**) were associated with the outcome. Results were shown for X and Z were independent (a) or correlated (b). K_{BC}, K_u, K_5 and K_w represent MiRKAT results from the Bray-Curtis kernel, unweighted UniFrac kernel, unweighted UniFrac kernel and generalized UniFrac kernels with $\alpha = 0.5$, respectively. K_{opt} represents the simulation results for optimal MiRKAT combining Bray-Curtis kernel, unweighted UniFrac kernel, weighted UniFrac kernel and generalized UniFrac kernel. MiSPU$_w$(2) and aMiSPU$_w$ represent MiSPU$_w$ test with $\gamma = 2$ and aMiSPU$_w$ summarizing $\gamma = 2, 3, \ldots, 8, \infty$, respectively. aMiSPU$_u$ and aMiSPU represent the test summarizing $\gamma = 2, 3, \ldots, 8, \infty$ and combing aMiSPU$_u$ and aMiSPU$_w$, respectively. $SPU_x(2)$ represents the SPU tests with the taxon proportions. Results were presented at $n = 100$.

Table S1: Empirical type I error for MiSPU and aMiSPU with binary outcome in scenario 1. Type I error rate was evaluated for scenarios in which covariates were independent with the OTUs ($X \perp\!\!\!\perp Z$) or related to the OTUs ($X \sim Z$) based on the 10,000 simulated datasets at $\alpha = 0.01$. *Inflated type I error rate.

	aMiSPU$_w$(2)	aMiSPU$_w$(∞)	aMiSPU$_w$	aMiSPU$_u$	aMiSPU
$X \perp\!\!\!\perp Z$, adjust X	0.010	0.011	0.011	0.009	0.009
$X \perp\!\!\!\perp Z$, no adjust X	0.010	0.012	0.010	0.009	0.010
$X \sim Z$, adjust X	0.006	0.005	0.006	0.009	0.008
$X \sim Z$, no adjust X	0.022*	0.038*	0.031*	0.010	0.024*
